# Connection to the Gay Community and Self-Appraisals Among Older Men Who Have Sex With Men

**DOI:** 10.1093/geroni/igaa057.3006

**Published:** 2020-12-16

**Authors:** Mark Brennan-Ing, Michael Plankey, Sabina Haberlen, Steven Meanley, Andre Brown, Deanna Ware, James Egan, Mackey Friedman

**Affiliations:** 1 Hunter College, CUNY, New York, New York, United States; 2 Georgetown University Medical Center, Washington, District of Columbia, United States; 3 Johns Hopkins University, Baltimore, Maryland, United States; 4 University of Pennsylvania, Philadelphia, Pennsylvania, United States; 5 University of Pittsburgh, Pittsburgh, Pennsylvania, United States

## Abstract

Men who have sex with men (MSM) report greater body dissatisfaction compared with heterosexual men, which increases with age. This may result from internalized gay community values regarding ideal physiques and youth. Using structural equation modeling, we examined these relationships among 1,118 MSM men age 40 and older from the Healthy Aging Study (M age=59.9 years/50.1% HIV+/69.8% non-Hispanic White). We hypothesized gay community attachment would be related to self-appraisals (body dissatisfaction/internalized ageism), and that this relationship would be mediated by developmental regulation strategies (physical activity/exercise intentions). The model fit the data well (RMSEA = .048, 90% CI 0.017-0.079). Contrary to our hypothesis, connection to the gay community was related to positive self-appraisals (-.40, p<.001), with significant indirect effects via regulation strategies (-.12, p<.002). Thus, gay community connections are related to positive self-appraisals in older GB men and enhance strategies supporting physical and psychological health. Implications for practice will be discussed.

